# Air pollution and cardiovascular mortality with over 25 years follow-up: A combined analysis of two British cohorts

**DOI:** 10.1016/j.envint.2016.12.004

**Published:** 2017-02

**Authors:** Hakim-Moulay Dehbi, Marta Blangiardo, John Gulliver, Daniela Fecht, Kees de Hoogh, Zaina Al-Kanaani, Therese Tillin, Rebecca Hardy, Nish Chaturvedi, Anna L Hansell

**Affiliations:** a MRC-PHE Centre for Environment and Health, Department of Epidemiology and Biostatistics, Imperial College London, London, W2 1PG, UK; b Institute of Cardiovascular Science, University College London, London, WC1E 7HB, UK; c MRC Unit for Lifelong Health and Ageing at UCL, University College London, London, WC1B 5JU, UK; d Imperial College Healthcare NHS Trust, London, UK; e Swiss Tropical and Public Health Institute, Basel, Switzerland; f University of Basel, Basel, Switzerland

**Keywords:** Follow-up studies, Environmental epidemiology, Particulate matter, Particles, Long-term exposure

## Abstract

**Background:**

Adverse effects of air pollution on cardiovascular disease (CVD) mortality are well established. There are comparatively fewer studies in Europe, and in the UK particularly, than in North America. We examined associations in two British cohorts with > 25 years of follow-up.

**Methods:**

Annual average NO_2_, SO_2_ and black smoke (BS) air pollution exposure estimates for 1991 were obtained from land use regression models using contemporaneous monitoring data. From the European Study of Cohorts and Air Pollution (ESCAPE), air pollution estimates in 2010–11 were obtained for NO_2_, NO_x_, PM_10_, PM_coarse_ and PM_2.5_. The exposure estimates were assigned to place of residence 1989 for participants in a national birth cohort born in 1946, the MRC National Study of Health and Development (NSHD), and an adult multi-ethnic London cohort, Southall and Brent Revisited (SABRE) recruited 1988–91. The combined median follow-up was 26 years. Single-pollutant competing risk models were employed, adjusting for individual risk factors.

**Results:**

Elevated non-significant hazard ratios for CVD mortality were seen with 1991 BS and SO_2_ and with ESCAPE PM_10_ and PM_2.5_ in fully adjusted linear models. Per 10 μg/m^3^ increase HRs were 1.11 [95% CI: 0.76–1.61] for BS, 1.05 [95% CI: 0.91–1.22] for SO_2_, 1.16 [95% CI: 0.70–1.92] for PM_10_ and 1.30 [95% CI: 0.39–4.34] for PM_2.5_, with largest effects seen in the fourth quartile of BS and PM_2.5_ compared to the first with HR 1.24 [95% CI: 0.91–1.61] and 1.21 [95% CI: 0.88–1.66] respectively. There were no consistent associations with other ESCAPE pollutants, or with 1991 NO_2_. Modelling using Cox regression led to similar results.

**Conclusion:**

Our results support a detrimental long-term effect for air pollutants on cardiovascular mortality.

## Highlights

• Adverse effects of air pollution on cardiovascular mortality are well established.
• There are comparatively fewer studies in the UK compared to North America.
• In two British cohort studies, we investigated associations between gases, particulates and cardiovascular mortality.
• Detrimental associations, non-statistically significant, between cardiovascular mortality and particulates were found.

## 1 **Introduction**

The association between long term exposure to outdoor air pollutants and cardiovascular disease [CVD] mortality has been investigated in multiple studies (Beelen et al., 2008, Beelen et al., 2014, Carey et al., 2013, Cesaroni et al., 2013, Crouse et al., 2015, Elliott et al., 2007, Filleul et al., 2005, Garcia et al., 2016, Hansell et al., 2016, Laden et al., 2006, Lepeule et al., 2012, Lipsett et al., 2011, Nafstad et al., 2004, Pope et al., 2002, Pope et al., 2004, Puett et al., 2011, Raaschou-Nielsen et al., 2012, Thurston et al., 2016a, Thurston et al., 2016b, Turner et al., 2016, Ueda et al., 2012, Yap et al., 2012). Most (Beelen et al., 2008, Crouse et al., 2015, Elliott et al., 2007, Filleul et al., 2005, Hansell et al., 2016, Laden et al., 2006, Lepeule et al., 2012, Nafstad et al., 2004, Pope et al., 2002, Pope et al., 2004, Yap et al., 2012), but not all (Puett et al., 2011, Ueda et al., 2012) studies with exposure information for the 1970s to 1990s, including the Harvard Six Cities (Laden et al., 2006) and American Cancer Society studies (Pope et al., 2002, Pope et al., 2004), have observed convincing evidence of an increased risk of CVD mortality with increasing exposure to air pollution, in particular with particulate matter (Beelen et al., 2008, Crouse et al., 2015, Filleul et al., 2005, Hansell et al., 2016, Laden et al., 2006, Lepeule et al., 2012, Pope et al., 2002, Pope et al., 2004) and with the gases NO_2_ and SO_2_ (Elliott et al., 2007, Nafstad et al., 2004). Most of the recent studies with more recent exposures support previously reported increased CVD risks associated with particulate matter (Cesaroni et al., 2013, Hansell et al., 2016, Lepeule et al., 2012, Lipsett et al., 2011, Thurston et al., 2016b, Turner et al., 2016) and NO_2_ (Raaschou-Nielsen et al., 2012), but other studies have weaker (Carey et al., 2013) or no association between particulate matter and CVD mortality (Beelen et al., 2014), the latter being the recently published European Study of Cohorts for Air Pollution Effects [ESCAPE] with an analysis of 367,383 individuals from 22 European cohorts (Beelen et al., 2014).

The objective of this study was to explore the relationship between air pollution and CVD mortality during a 25-year follow-up period in a UK setting, using both air pollution estimates modelled incorporating air pollution monitoring data from the 1990s and air pollution estimates from the ESCAPE study (Beelen et al., 2013, Eeftens et al., 2012). We combined two long-running British cohorts, one with wide geographical representation, but with lower exposure to air pollution [the Medical Research Council [MRC] National Survey of Health and Development [NSHD] (Kuh et al., 2011, Wadsworth et al., 2006)], and one geographically discrete cohort recruited from North-West London [the Southall And Brent REvisited [SABRE] study (Tillin et al., 2012)] with relatively high air pollution exposure levels.

## 2 Methods

### 2.1 Cohort data

The NSHD originally consisted of 5362 individuals recruited from all singleton births occurring in a single week in March 1946. Since birth, NSHD participants have been followed up at regular intervals (Kuh et al., 2011, Wadsworth et al., 2006) and the cohort responding at the data collection at age 60–64 remains broadly representative of the general population, despite some loss to follow-up for more deprived groups (Stafford et al., 2013). SABRE is a tri-ethnic [European, South Asian and African-Caribbean, the latter two groups being first generation migrants] population-based cohort of 4857 men and women from North-West London. Participants were recruited from primary care lists and factory workforces and were aged 40 to 69 years at the study start in 1989–91 (Tillin et al., 2012). The year 1989 was chosen as ‘baseline’ for our study as it was the year in which SABRE began and also a follow-up year for the NSHD when research nurses visited participants in their own homes.

Cohort participants are flagged on the NHS central register so that all deaths are identified and CVD mortality diagnosed as International Classification of Disease-Ninth Edition [ICD-9] codes 390–459 and ICD-Tenth Edition [ICD-10] chapter I codes. The follow-up for mortality was until the end of 2014 for NSHD and November 2015 for SABRE.

Additionally, in SABRE, an analysis of the combined endpoint of CVD mortality and morbidity was possible, from baseline to end of 2011 (Tillin et al., 2013). The objective was to investigate whether the association between air pollution and CVD is modified by the addition of non-fatal events to the endpoint. Three sources of information were used to confirm a non-fatal CVD event during follow-up: 1) primary care data review for a definite or probable diagnosis of myocardial infarction or acute coronary syndrome or stroke; 2) Hospital Episode Statistics for non-fatal coronary heart disease or stroke events; 3) participant report of physician-diagnosed stroke with duration of symptoms in excess of 24 h.

Confounders at baseline chosen a priori were age, gender, ethnicity, smoking status [current, ex-, non-], individual socio-economic status [manual and non-manual employment using the Registrar General's social classification (Anon., 1980)], 1991 Carstairs index of area-level deprivation [which is an index of deprivation at the area-level based on four census variables: non car ownership, low social class, overcrowding and unemployment (Carstairs and Morris, 1991)], CVD diagnosis [from medical history questionnaire in both cohorts, and additionally in SABRE, using primary care record review and hospital episode statistics] and diabetes status [from self-reported physician diagnosis in both cohorts, and additionally in SABRE, use of anti-diabetic medication or positive glucose tolerance test]. The ethnicity of SABRE participants was self-assigned, confirmed by the country of birth of both parents. NSHD participants were all Caucasian, as representative of the British-born population in 1946 which predated major immigration flows.

### 2.2 Air pollution concentration estimates

Two sets of air pollution estimates were available. Firstly, concentration estimates of sulphur dioxide [SO_2_], black smoke [BS] and nitrogen dioxide [NO_2_] were available for 1991, based on contemporaneous air pollution monitoring data. Secondly, from ESCAPE, estimates of NO_2_, NO_x_, PM_10_, PM_coarse_ and PM_2.5_ concentrations were available, based on monitoring data in 2010–11 and non-extrapolated.

We used these two sets of estimates to take into account different aspects of air pollution. The effect of gases (NO_2_, NO_x_ and SO_2_) was investigated alongside the effect of particulates, which we approached using both BS and PM. BS is a measure of particulates dominated by elemental carbon particles; BS is largely insensitive to secondary inorganic aerosols, but both contribute to PM.

#### 2.2.1 1991 air pollution estimates

Estimates of SO_2_, BS and NO_2_ concentrations were available for the year 1991 and assigned to residence at baseline (postcode at study start of SABRE in 1989–1991 and address for follow-up year of NSHD in 1989). Land-use regression [LUR] techniques were employed to model concentrations of these air pollutants, based on contemporaneous monitoring data from the national air quality monitoring network for BS and SO_2_ (Gulliver et al., 2011) and a national NO_2_ network for NO_2_ (Gulliver et al., 2016). For BS and SO_2_, 966 and 825 monitoring sites were used respectively and 451 monitoring sites for NO_2_. Great-Britain-wide air pollution maps were produced with a resolution of 100 m × 100 m for BS and SO_2_, and 200 m × 200 m for NO_2_. The X-Y coordinates of the residence of participants were overlaid with these maps to assign concentration estimates. Model building employed 80% of the sites for BS and SO_2_, and 75% of the sites for NO_2_. The remaining sites were retained for model validation, and hold-out r^2^ values of 0.34, 0.31 and 0.62 were obtained for BS, SO_2_ and NO_2_ respectively.

These concentration estimates are referred to as contemporaneous 1991 estimates in the rest of the text.

#### 2.2.2 ESCAPE air pollution estimates

The LUR models developed for the ESCAPE project were employed to assign estimates of exposure to NO_2_, NO_x_, PM_10_, PM_coarse_ and PM_2.5_. The details of the models have been described elsewhere (Beelen et al., 2013, Eeftens et al., 2012). For 20 European study areas, measurements were taken at 20–40 sites for PM and 40–80 sites for NO_2_ and NO_x_. In England, annual average concentrations of these air pollutants were monitored in London, the Thames valley and the Manchester area between January 2010 and January 2011. The median model explained variances (r^2^) over the study areas of the LUR models were 82% for NO_2_, 78% for NO_x_, 77% for PM_10_, 68% for PM_coarse_ and 71% for PM_2.5_.

In both cohorts, ESCAPE estimates for 2010–11 were used to estimate past exposures as geographical variability in air pollution concentrations have been shown to be relatively stable over time (Gulliver et al., 2016), which was supported in ESCAPE analyses (Beelen et al., 2014) that found similar results using back-extrapolated as 2010–11 estimated concentrations.

We refer to these air pollutants as the ESCAPE 2010–11 estimates in the rest of the text.

### 2.3 Statistical methods

Competing risk hazards regression models (Jason and Gray, 1999) were used to study the effect of air pollutants on incident fatal CVD events to allow for the presence of competing events, which in this case is death from cause other than CVD. The outcome of the survival model was time in months from baseline until death from CVD or censoring. Death from other causes than CVD was considered as a competing event. Participants without death notification were censored at the end of the follow-up time [December 2014 for NSHD, November 2015 for SABRE]. In a sensitivity analysis, Cox modelling was used to verify that results were not influenced by the analysis method.

Single-pollutant models were employed, with air pollutants fitted as both continuous variables and as quartiles to explore the possibility of non-linear associations. Moreover, two two-pollutant models were considered for the 1991 air pollution estimates: BS + NO_2_ and SO_2_ + NO_2_. Other combinations were not considered due to high correlation between air pollutants. Four levels of confounder adjustment were employed:

• M1: the basic model, adjusted for the cohort to which the participant belongs;
• M2: M1 plus age and gender;
• M3: M2 plus socio-economic status and 1991 Carstairs index;
• M4: M3 plus diabetes, smoking status, baseline CVD and ethnicity.

Effect modification of air pollution, using interaction terms, was studied by gender, diabetes status, smoking status, type of employment and ethnicity.

Plots of the Schoenfeld residuals against time, for each covariate, were examined to verify that the proportional hazard [PH] assumption of the model was not violated (Schoenfeld, 1982). There were no signs of violations of assumptions.

All analyses were performed in the R environment for statistical computing, version 3.1.2 [R Foundation for Statistical Computing, Vienna, Austria].

## 3 Results

### 3.1 Participants

**Fig. 1:**
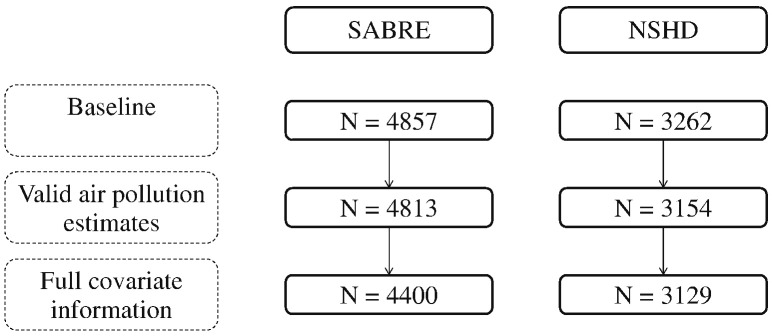
Flowchart of study participants (valid air pollution estimates based on 1991 estimates of NO_2_, SO_2_ and BS).

There were 4400 participants from SABRE and 3129 from the NSHD included in the study (Fig. 1), with a median follow-up of 26 years. Participants' characteristics are presented in Table 1. SABRE participants were on average nine years older than NSHD participants at baseline. By design, approximately a quarter of SABRE participants were women, while this proportion was 50% in NSHD. More than half of SABRE was non-smokers, compared to 30% in NSHD. In contrast, diabetes prevalence, at 14%, was greater in SABRE than in NSHD at 1%. By the end of follow-up, 1402/4400 [32%] and 337/3139 [11%] of SABRE and NSHD participants respectively had died, 519 [37%] and 91 [27%] of these deaths respectively were due to CVD.

### 3.2 Air pollution assessment

**Fig. 2:**
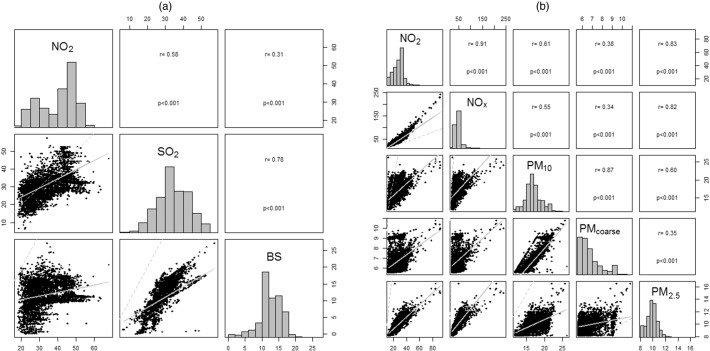
Histograms of air pollutants for the combined SABRE + NSHD cohort (diagonal), scatter plots (straight line representing the linear fit between the two air pollutants; dashed line the identity line) (below the diagonal) and correlations between air pollutants (above the diagonal). (a): contemporaneous 1991 estimates. (b): ESCAPE 2010–11 estimates.

SABRE participants were exposed to higher concentration levels than NSHD participants of BS [difference in median: 1.6 μg/m^3^], SO_2_ [difference in median: 10.5 μg/m^3^], NO_2_ [difference in median: 16.4 μg/m^3^] according to the contemporaneous 1991 estimates, and to higher concentrations levels of PM_10_ [difference in median: 1.5 μg/m^3^], PM_coarse_ [difference in median: 0.7 μg/m^3^] and PM_2.5_ [difference in median: 0.4 μg/m^3^] according to the ESCAPE 2010–11 estimates [Table 1 and Appendix Fig. 1]. The distributions of concentration levels were narrower in SABRE than in NSHD, reflecting the smaller geographical area of the SABRE cohort in North-West London, compared to the NSHD living throughout Britain. NO_2_ exposure estimates fell between 1991 and 2010–11 by approximately 8 μg/m^3^ in NSHD and 16 μg/m^3^ in SABRE. Some air pollutants were highly correlated [Fig. 2]. For the 1991 estimates, the correlation coefficient for BS and SO_2_ was 0.79, but for BS and NO_2_ it was 0.28. For ESCAPE 2010–11 estimates, the correlation was 0.91 between NO_2_ and NO_x_, 0.83 between NO_2_ and PM_2.5_ and 0.86 between PM_10_ and PM_coarse_.

### 3.3 Analytic analyses

**Fig. 3:**
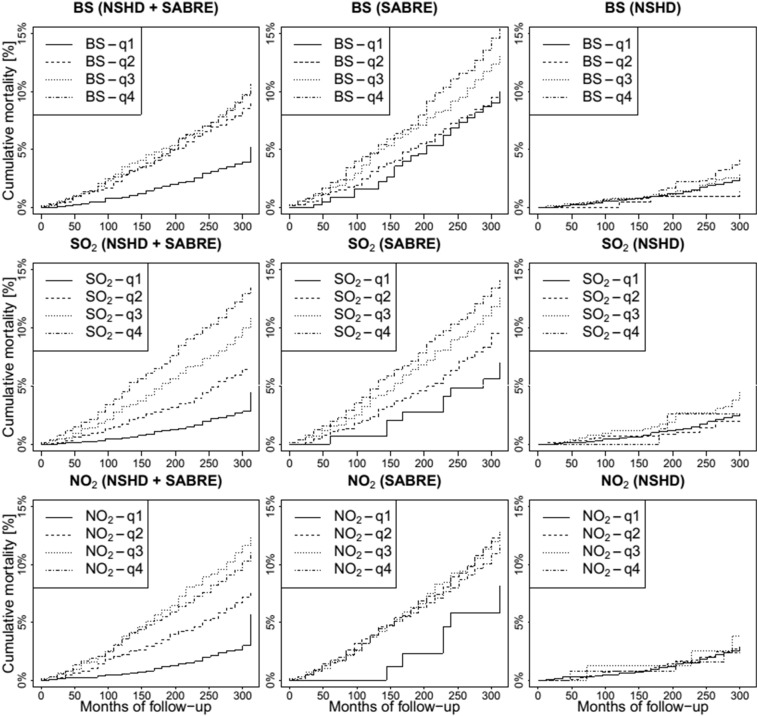
Cumulative CVD mortality incidence by quartile of BS, SO_2_ and NO_2_. The first column corresponds to the combination of the two cohorts.

Cumulative incidence of CVD mortality showed separation across the quartiles of air pollutants [Fig. 3] that was greater in SABRE than in NSHD, reflecting the much larger number of events in the SABRE cohort.

In the basic model, higher levels of SO_2_ and BS, but not NO_2_, were associated with higher rates of CVD mortality [Fig. 3 and Table 2]. The BS and SO_2_ associations became weaker and non-significant on multivariable adjustment [in particular after adjustment for socio-economic status and 1991 Carstairs index] but remained elevated. In the fully-adjusted model, the hazard ratio [HR] for BS was 1.11 [95% CI: 0.76–1.61] and SO_2_ 1.05 [95% CI: 0.91–1.22] per increase of 10 μg/m^3^. Analysis by quartiles suggested potential non-linearity, with largest associations in the highest BS quartile, fully adjusted HR 1.24 [95% CI: 0.91–1.69] for highest versus lowest quartile. The associations with BS and SO_2_ were not affected by further adjustment for NO_2_, either as a continuous variable or as quartiles [Supplemental Table 1].

There were no statistically significant associations detected using the ESCAPE 2010–11 estimates [Table 3]. However, for PM_2.5_ and PM_10_, the HRs were markedly elevated but statistically non-significant in unadjusted models and remained elevated, although to a lesser extent, in fully-adjusted models. In fully-adjusted analyses, the HR for PM_2.5_ was 1.30 [95% CI: 0.39–4.34] and for PM_10_ was 1.16 [95% CI: 0.70–1.92] per increase of 10 μg/m^3^. The second quartile of PM_2.5_ was statistically significantly elevated compared to the first, with no consistent pattern of increased risk with increased exposure seen across PM_2.5_ quartiles so this may represent a chance finding.

In an additional analysis in SABRE using the combination of incident CVD mortality and CVD morbidity, findings were comparable to those in the combined SABRE and NSHD mortality analyses, with positive non-significant associations seen for 1991 BS and SO_2_ but no associations for 1991 NO_2_ or ESCAPE pollutants [Supplemental Tables 2 and 3].

No effect modification by gender, diabetes status, smoking status, type of employment were found in any analyses [results not shown]. Results did not depend on the analysis methods as Cox models provided results that were similar to competing risk models [Supplemental Tables 4 and 5]. As history of diabetes and CVD may be in the causal between air pollution of cardiovascular mortality, we removed the adjustments for these two factors from the fully-adjusted models in a sensitivity analysis. We observed that this did not influence the results [Supplemental Table 6].

## 4 Discussion

In this combined analysis of two British cohort studies followed-up for > 25 years, we found non-statistically significant increases in CVD mortality risk associated with exposure to SO_2_ and particulate measures (BS, PM_10_, PM_2.5_).

Our study in the UK contributes to the body of scientific evidence collected on cardiovascular mortality risk of air pollution, as most of the studies took place in North America (Crouse et al., 2015, Garcia et al., 2016, Laden et al., 2006, Lepeule et al., 2012, Lipsett et al., 2011, Pope et al., 2002, Pope et al., 2004, Puett et al., 2011, Thurston et al., 2016a, Thurston et al., 2016b, Turner et al., 2016). There are comparatively fewer studies in Europe (Beelen et al., 2008, Beelen et al., 2014, Cesaroni et al., 2013, Filleul et al., 2005, Nafstad et al., 2004, Raaschou-Nielsen et al., 2012), in the UK (Carey et al., 2013, Elliott et al., 2007, Hansell et al., 2016, Yap et al., 2012) and in other regions of the world (Ueda et al., 2012). In the UK, our study adds to the currently limited evidence for a detrimental effect of particulates on CVD mortality, which has been reported in some (Elliott et al., 2007, Hansell et al., 2016, Yap et al., 2012) but not all studies (Carey et al., 2013).

Recent meta-analyses, in terms of cerebrovascular events (Scheers et al., 2015), hypertension (Cai et al., 2016) and markers of subclinical disease (Akintoye et al., 2016, Provost et al., 2015), point towards a detrimental effect of air pollution. This is coherent with findings for CVD mortality, where the majority of past studies report detrimental associations between BS and SO_2_, with HRs varying between 1.03 and 1.11 (Beelen et al., 2008, Carey et al., 2013, Filleul et al., 2005, Hansell et al., 2016, Nafstad et al., 2004, Yap et al., 2012) per increase of 10 μg/m^3^ in these air pollutants. Our adjusted HRs were 1.11 [95% CI: 0.76–1.61] for BS and 1.05 [95% CI: 0.91–1.22] for SO_2_ per increase of 10 μg/m^3^, which is in line with previously reported estimates. For PM_10_ and PM_2.5_, the majority of past studies also report detrimental associations with CVD mortality, with HRs for PM_2.5_ varying between 1.03 and 1.13 per 10 μg/m^3^ (Cesaroni et al., 2013, Crouse et al., 2015, Thurston et al., 2016a, Turner et al., 2016) and, for PM_10_ in the large study of the UK Longitudinal Study (Hansell et al., 2016), an OR of 1.12 [95% CI: 1.01 to 1.25]. Our HRs, per increase of 10 μg/m^3^, for PM_2.5_ and PM_10_ (using ESCAPE estimates) were 1.30 [95% CI: 0.39–4.34] and 1.16 [95% CI: 0.70–1.92] respectively. This is consistent with these previously reported estimates, as well as with the ESCAPE project in which elevated non-significant HRs for PM_2.5_ of 1.21 [95% CI: 0.87–1.69] per increase of 5 μg/m^3^ and PM_10_ of 1.22 [95% CI: 0.91–1.63] per 10 μg/m^3^ were reported. In our study, we did not find significant effect modifications and were unable to replicate subgroup associations previously suggested, in particular for people with diabetes (Brook et al., 2010). However, this may relate to inherent lack of power of statistical tests for interaction and relatively small study size.

While the majority of studies relating to historical exposures have found a clear statistically significant effect of air pollution on CVD risk (Beelen et al., 2008, Cesaroni et al., 2013, Crouse et al., 2015, Elliott et al., 2007, Filleul et al., 2005, Hansell et al., 2016, Laden et al., 2006, Lepeule et al., 2012, Lipsett et al., 2011, Nafstad et al., 2004, Pope et al., 2002, Pope et al., 2004, Raaschou-Nielsen et al., 2012, Thurston et al., 2016b, Turner et al., 2016, Yap et al., 2012), our study found non-statistically significant detrimental associations. This might be related to differences in follow-up time and statistical power, to changes in chemical constituents and degree of exposure to air pollution over time and between areas, or because of improved treatments for CVD including CVD risk factors. Firstly in terms of statistical power, a study of more than one million adults in Rome (Cesaroni et al., 2013) reported a HR for CVD mortality of 1.06 [95% CI: 1.04–1.08] per 10 μg/m^3^ increase in PM_2.5_. This effect size is modest in magnitude; therefore many events and consequently a large sample size are required to reach statistical significance. Secondly with respect to exposure, concentrations differ between areas and over time and while the exposure-response relationship is often considered as linear, this might differ at different concentrations. For example, in the Longitudinal Study in the UK (Pope et al., 2002), mean BS levels fell from 42.7 μg/m^3^ to 11.8 μg/m^3^ over the twenty years 1971–1991. Thirdly, with regards to improved treatments for CVD, the ESCAPE study reported detrimental but non-significant associations between PM_2.5_ and PM_10_ and CVD mortality (Beelen et al., 2014) but significant associations with incidence of acute coronary events (Cesaroni et al., 2014), which may support a hypothesis that improved treatments are resulting in lower case-fatality, thereby influencing the exposure-response relationship. Finally, differential exposure assessment (i.e. different levels of exposure misclassification) and/or confounders assessment might also partly explain the difference between our non-statistically significant results and results from other studies. Validation r^2^ values of the LUR models for BS and SO_2_ were low, which will have resulted in exposure misclassification and potential impact on the associations, particularly for SO_2_. However, we have demonstrated associations with all cause and cardiovascular mortality using the same model estimates in another but much larger cohort (Hansell et al., 2016).

The strengths of our analysis are the following. Firstly, by combining two cohorts with different geographical locations, we increased the range of air pollution covered. Moreover, by combining SABRE and NSHD, we added diversity in terms of ethnicity. In our combined sample, 30% of the participants are not European (21% are of South Asian origin and 9% of African Caribbean origin). Secondly, because of the wealth of information contained in these cohorts, we could adjust our analyses for multiple important possible confounders, in particular age, gender, individual and area-level social economic status, baseline CVD, ethnicity, smoking and diabetes status. Nevertheless, we cannot exclude potentially incomplete adjustment for confounding. Thirdly, we used modelled estimates of air pollution concentrations in 1991 derived from LUR models developed using measurements for 1991 from the national monitoring network and evaluated with a hold-out set of measurement (Gulliver et al., 2011, Gulliver et al., 2016) rather than relying on sparse monitoring networks or back extrapolation. Nevertheless, exposure misclassification is a limitation of modelled air pollutants in epidemiological studies, particularly as this is usually assigned to residence rather than the specific activity patterns of the person (Brown et al., 2009). Further, we used air pollution concentrations at specific time-points assuming that spatial contrasts stayed the same [as in the ESCAPE study of CVD mortality (Beelen et al., 2014)] and that participants did not change their address during the follow-up period as we did not have a full residential history for each participant. For NO_2_, we noted that the effect of two different estimation periods did not change the results markedly by comparing the results using the contemporaneous 1991 estimate and the ESCAPE 2010–11 estimate: contemporaneous 1991 estimate HR 0.97 (0.81–1.16) and ESCAPE 2010–11 estimates HR 1.03 (0.88–1.20).

In conclusion, we found a non-statistically significant detrimental association between particulate measures (BS, PM_10_, PM_2.5_) and CVD mortality, which is consistent with findings in other recently published studies. Further research into cardiovascular effects of air pollution is needed, in particular at the clinical and sub-clinical levels to better understand the pathways by which air pollution may promote CVD.

## Figures and Tables

**Table 1:** Demographic, health and air pollution exposure at baseline for SABRE and NSHD cohorts individually and combined, and numbers of deaths by the end of follow-up. * The NSHD is predominantly white British as they are representative of the British-born population in 1946 which predated major immigration flows.

	NSHD	SABRE	SABRE + NSHD
n	3129	4400	7529

*Participants' characteristics*			
Age [years] - mean (SD)	43.00 (0.0)	52.33 (6.9)	48.45 (7.0)
Sex = female (%)	1558 (49.8)	1033 (23.5)	2591 (34.4)
Smoking (%)			
Current smoker	937 (29.9)	1038 (23.6)	1975 (26.2)
Ex-smoker	1284 (41.0)	1027 (23.3)	2311 (30.7)
Non-smoker	908 (29.0)	2335 (53.1)	3243 (43.1)
Diabetes (%)	34 (1.1)	601 (13.7)	635 (8.4)
Job = non-manual (%)	1700 (54.3)	1394 (31.7)	3094 (41.1)
Ethnicity (%)			
African Caribbean	0 (0.0)	660 (15.0)	660 (8.8)
European	3129 (100.0)*	2157 (49.0)	5286 (70.2)
South Asian	0 (0.0)	1583 (36.0)	1583 (21.0)
CVD at baseline (%)	111 (3.5)	386 (8.8)	497 (6.6)

*Mortality*			
Number of deaths (%)	337 (10.8)	1402 (31.8)	1739 (23.1)
African Caribbean	0 (0.0)	160 (3.6)	160 (2.1)
European	337 (10.8)	784 (17.8)	1121 (14.9)
South Asian	0 (0.0)	458 (10.4)	458 (6.1)
Number of CVD deaths (%)	91 (2.9)	519 (11.7)	610 (8.1)
African Caribbean	0 (0.0)	51 (1.1)	51 (0.7)
European	91 (2.9)	265 (6.0)	356 (4.7)
South Asian	0 (0.0)	203 (4.6)	203 (2.7)

*Air pollution exposure*			
Contemporaneous 1991estimates			
NO_2_ [μg/m^3^] - median (IQR)	29.44 (25.47–35.39)	45.84 (44.91–48.77)	44.24 (30.85–46.19)
SO_2_ [μg/m^3^] - median (IQR)	27.97 (24.07–31.97)	38.45 (32.79–44.25)	32.96 (28.77–41.25)
BS [μg/m^3^] - median (IQR)	11.27 (8.81–14.70)	12.91 (11.35–14.88)	12.48 (11.22–14.88)
ESCAPE 2010–11 estimates			
NO_2_ [μg/m^3^] - median (IQR)	22.50 (17.59–27.27)	30.70 (28.40–32.60)	28.80 (21.79–31.80)
NO_x_ [μg/m^3^] - median (IQR)	37.08 (28.53–45.37)	46.90 (40.90–51.70)	43.50 (35.29–50.00)
PM_10_ [μg/m^3^] - median (IQR)	15.80 (14.87–16.72)	17.30 (16.40–18.50)	16.70 (15.67–17.90)
PM_coarse_ [μg/m^3^] - median (IQR)	6.04 (5.79–6.55)	6.70 (6.30–7.30)	6.40 (6.00–7.10)
PM_2.5_ [μg/m^3^] - median (IQR)	9.63 (8.85–10.25)	10.00 (9.60–10.50)	9.90 (9.40–10.40)

**Table 2:** Single-pollutant hazard ratios (HR) and 95% confidence intervals (CI), per increase of 10 μg/m^3^ for the continuous variables, between air pollution exposure to BS, SO_2_, NO_2_ (contemporaneous 1991 estimates) and CVD mortality (1989–2015). M1: model adjusted only for cohort to which the participant belongs. M2: M1 + age, gender. M3: M2 + type of employment, 1991 Carstairs index. M4 (fully-adjusted model): M3 + diabetes, smoking status, ethnicity, baseline CVD.

	# Events/n (%)	M1	M2	M3	M4
Continuous					
NO_2_	610/7529 (8.1%)	1.03 (0.90 to 1.18)	1.06 (0.91 to 1.23)	0.94 (0.79 to 1.12)	0.97 (0.81 to 1.16)
SO_2_	610/7529 (8.1%)	1.27 (1.13 to 1.42)	1.19 (1.05 to 1.34)	1.07 (0.94 to 1.23)	1.05 (0.91 to 1.22)
BS	610/7529 (8.1%)	1.66 (1.19 to 2.32)	1.44 (1.02 to 2.04)	1.16 (0.81 to 1.65)	1.11 (0.76 to 1.61)

Quartiles					
NO_2_-q1:[18.2–31.4]	62/1974 (3.1%)	1	1	1	1
NO_2_-q2:(31.4–44.4]	142/1909 (7.4%)	0.99 (0.7 to 1.39)	0.93 (0.65 to 1.33)	0.76 (0.51 to 1.12)	0.75 (0.50 to 1.11)
NO_2_-q3:(44.4–46.6]	224/1868 (12.0%)	1.24 (0.86 to 1.79)	1.10 (0.75 to 1.62)	0.86 (0.56 to 1.32)	0.86 (0.56 to 1.32)
NO_2_-q4:(46.6–67.9)	182/1778 (10.2%)	1.07 (0.74 to 1.55)	1.05 (0.71 to 1.56)	0.81 (0.53 to 1.26)	0.87 (0.56 to 1.34)
SO_2_-q1:[6.5–29.2]	60/1974 (3.0%)	1	1	1	1
SO_2_-q2:(29.2–33.0]	113/1820 (6.2%)	1.02 (0.73 to 1.44)	1.04 (0.73 to 1.47)	0.82 (0.55 to 1.20)	0.85 (0.58 to 1.25)
SO_2_-q3:(33.0–41.2]	213/2069 (10.3%)	1.52 (1.08 to 2.12)	1.35 (0.95 to 1.91)	1.10 (0.76 to 1.61)	1.08 (0.74 to 1.57)
SO_2_-q4:(41.2–57.3)	224/1666 (13.4%)	1.66 (1.17 to 2.34)	1.43 (1.00 to 2.05)	1.01 (0.67 to 1.51)	1.01 (0.67 to 1.51)
BS-q1:[0.08–11.2]	82/2000 (4.1%)	1	1	1	1
BS-q2:(11.2–12.5]	151/1776 (8.6%)	0.96 (0.71 to 1.29)	0.94 (0.69 to 1.27)	0.92 (0.68 to 1.26)	0.98 (0.71 to 1.34)
BS-q3:(12.5–14.9]	217/2182 (9.9%)	1.27 (0.96 to 1.69)	1.15 (0.86 to 1.53)	1.01 (0.75 to 1.36)	1.02 (0.75 to 1.39)
BS-q4:(14.9–27.2]	160/1581 (10.1%)	1.54 (1.16 to 2.06)	1.42 (1.05 to 1.90)	1.23 (0.91 to 1.66)	1.24 (0.91 to 1.69)

**Table 3:** Single-pollutant hazard ratios (HR) and 95% confidence intervals (CI), per increase of 10 μg/m^3^ for the continuous variables, between ESCAPE 2010–11 air pollution estimates of NO_2_, NO_x_, PM_10_, PM_coarse_, PM_2.5_ and CVD mortality (1989–2015). M1: model adjusted only for cohort to which the participant belongs. M2: M1 + age, gender. M3: M2 + type of employment, 1991 Carstairs index. M4 (fully-adjusted model): M3 + diabetes, smoking status, ethnicity, baseline CVD.

	# Events/n (%)	M1	M2	M3	M4
Continuous					
NO_2_	422/6454 (6.5%)	1.06 (0.93 to 1.20)	1.10 (0.96 to 1.26)	1.00 (0.86 to 1.17)	1.03 (0.89 to 1.20)
NO_x_	422/6454 (6.5%)	1.01 (0.96 to 1.07)	1.03 (0.97 to 1.09)	1.00 (0.93 to 1.07)	1.01 (0.94 to 1.07)
PM_10_	422/6454 (6.5%)	1.28 (0.82 to 2.01)	1.36 (0.86 to 2.15)	1.12 (0.68 to 1.85)	1.16 (0.70 to 1.92)
PM_coarse_	422/6454 (6.5%)	1.02 (0.40 to 2.60)	1.12 (0.43 to 2.91)	0.82 (0.29 to 2.33)	0.89 (0.31 to 2.55)
PM_2.5_	422/6454 (6.5%)	1.52 (0.52 to 4.46)	2.10 (0.69 to 6.44)	1.15 (0.35 to 3.81)	1.30 (0.39 to 4.34)

Quartiles					
NO_2_-q1:[12.9–21.8]	61/1614 (3.8%)	1	1	1	1
NO_2_-q2:(21.8–28.8]	89/1628 (5.5%)	1.05 (0.76 to 1.47)	1.08 (0.78 to 1.50)	0.96 (0.69 to 1.34)	0.95 (0.68 to 1.34)
NO_2_-q3:(28.8–31.8]	148/1643 (9.0%)	1.23 (0.90 to 1.68)	1.18 (0.86 to 1.61)	0.99 (0.72 to 1.36)	1.03 (0.74 to 1.43)
NO_2_-q4:(31.8–91.8]	124/1569 (8.0%)	1.17 (0.84 to 1.61)	1.24 (0.90 to 1.72)	1.00 (0.71 to 1.41)	1.06 (0.75 to 1.50)
NO_x_-q1:[19.7–35.3]	72/1614 (4.5%)	1	1	1	1
NO_x_-q2:(35.3–43.5]	98/1633 (6.0%)	0.93 (0.68 to 1.28)	0.91 (0.67 to 1.25)	0.82 (0.60 to 1.13)	0.83 (0.60 to 1.14)
NO_x_-q3:(43.5–50.0)	125/1593 (7.8%)	1.01 (0.74 to 1.36)	0.99 (0.73 to 1.34)	0.83 (0.61 to 1.13)	0.84 (0.61 to 1.16)
NO_x_-q4:(50.0–242.0)	127/1614 (7.9%)	1.07 (0.79 to 1.45)	1.11 (0.82 to 1.50)	0.90 (0.65 to 1.24)	0.96 (0.69 to 1.32)
PM_10_-q1:[11.8–15.7]	62/1614 (3.8%)	1	1	1	1
PM_10_-q2:(15.7–16.7]	97/1665 (5.8%)	1.12 (0.82 to 1.55)	1.14 (0.83 to 1.57)	1.06 (0.77 to 1.47)	1.04 (0.75 to 1.44)
PM_10_-q3:(16.7–17.9]	136/1584 (8.6%)	1.34 (0.98 to 1.83)	1.34 (0.98 to 1.83)	1.15 (0.83 to 1.59)	1.15 (0.83 to 1.60)
PM_10_-q4:(17.9–26.3]	127/1591 (8.0%)	1.22 (0.89 to 1.67)	1.25 (0.91 to 1.72)	1.06 (0.76 to 1.48)	1.06 (0.76 to 1.48)
PM_coarse_-q1:[5.6–6.0]	66/1751 (3.8%)	1	1	1	1
PM_coarse_-q2:(6.0–6.4]	101/1485 (6.8%)	1.37 (1.00 to 1.87)	1.30 (0.95 to 1.78)	1.22 (0.89 to 1.67)	1.16 (0.84 to 1.59)
PM_coarse_-q3:(6.4–7.1]	138/1636 (8.4%)	1.43 (1.06 to 1.93)	1.45 (1.08 to 1.95)	1.19 (0.86 to 1.64)	1.23 (0.89 to 1.70)
PM_coarse_-q4:(7.1–10.8)	117/1582 (7.4%)	1.25 (0.91 to 1.70)	1.24 (0.91 to 1.69)	1.07 (0.77 to 1.48)	1.09 (0.79 to 1.51)
PM_2.5_-q1:[8.2–9.4]	70/1688 (4.1%)	1	1	1	1
PM_2.5_-q2:(9.4–9.9]	135/1577 (8.6%)	1.51 (1.13 to 2.02)	1.49 (1.11 to 2.00)	1.40 (1.04 to 1.87)	1.37 (1.01 to 1.84)
PM_2.5_-q3:(9.9–10.4]	104/1631 (6.4%)	1.08 (0.80 to 1.47)	1.07 (0.79 to 1.45)	0.95 (0.70 to 1.29)	0.98 (0.72 to 1.34)
PM_2.5_-q4:(10.4–16.5]	113/1558 (7.3%)	1.31 (0.96 to 1.77)	1.37 (1.01 to 1.85)	1.17 (0.86 to 1.60)	1.21 (0.88 to 1.66)

## Appendix A Supplementary data

Supplementary material Image 1
