# Supplementary material for: Air pollution and cardiovascular mortality with over 25 years follow-up: A combined analysis of two British cohorts
Source: Environ Int. 2017 Feb;99:275–81. doi: 10.1016/j.envint.2016.12.004 (PMC5292102; doi:10.1016/j.envint.2016.12.004)
Supplement: Supplementary file 1 — Supplementary material [file mmc1.docx]

**Supplemental material**

Air pollution and cardiovascular mortality with over 25 years follow-up: A combined analysis of two British cohorts

Hakim-Moulay Dehbi, Marta Blangiardo, John Gulliver, Daniela Fecht, Kees de Hoogh, Zaina Al-Kanaani, Therese Tillin, Rebecca Hardy, Nish Chaturvedi, Anna L Hansell

Supplemental Figure 1: Histograms of air pollutants for SABRE and NSHD participants. (a): contemporaneous 1991 estimates. (b): ESCAPE 2010-11 estimates


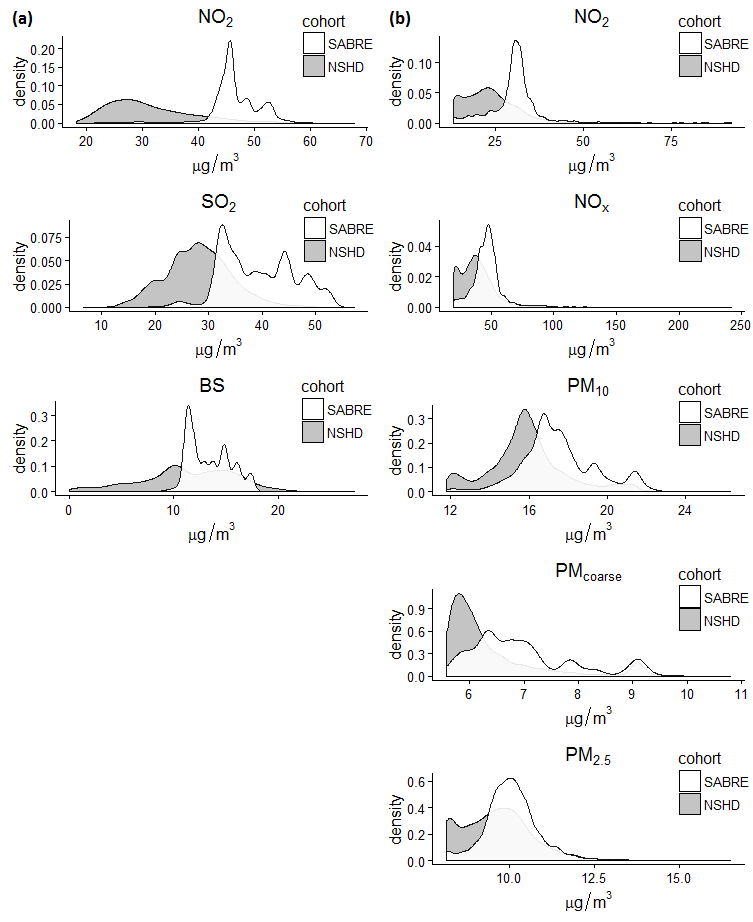


Supplemental Table 1: Two-pollutant hazard ratios (HR) and 95% confidence intervals (CI), per increase of 10 µg/m^3^ for the continuous variables, between air pollution exposure to BS + NO2 and SO2 + NO2 and CVD mortality (1989-2015). M1: model adjusted only for cohort to which the participant belongs. M4 (fully-adjusted model): M2 + age, gender, diabetes, smoking status, ethnicity, type of employment, 1991 Carstairs index and baseline CVD.

|  | **# events / n (%)** | **M1: model adjusted for cohort only** | **M4: fully-adjusted model** |
| --- | --- | --- | --- |
| **BS + NO_2_** |  |  |  |
| **Continuous** |  |  |  |
| NO2 | 610 / 7529 (8.1%) | 1.03 (0.90 to 1.19) | 0.97 (0.81 to 1.16) |
| BS | 610 / 7529 (8.1%) | 1.67 (1.19 to 2.33) | 1.11 (0.76 to 1.60) |
|  |  |  |  |
| **Quartiles** |  |  |  |
| NO_2_-q1:[18.2-31.4] | 62 / 1974 (3.1%) | 1 | 1 |
| NO_2_-q2:(31.4-44.4] | 142 / 1909 (7.4%) | 0.99 (0.70 to 1.40) | 0.80 (0.54 to 1.18) |
| NO_2_-q3:(44.4-46.6] | 224 / 1868 (12.0%) | 1.15 (0.78 to 1.71) | 0.87 (0.56 to 1.34) |
| NO_2_-q4:(46.6-67.9) | 182 / 1778 (10.2%) | 1.13 (0.77 to 1.66) | 0.92 (0.59 to 1.43) |
| BS-q1:[0.08-11.2] | 82 / 2000 (4.1%) | 1 | 1 |
| BS-q2:(11.2-12.5] | 151 / 1776 (8.6%) | 0.96 (0.71 to 1.28) | 0.99 (0.73 to 1.34) |
| BS-q3:(12.5-14.9] | 217 / 2182 (9.9%) | 1.27 (0.96 to 1.69) | 1.06 (0.79 to 1.44) |
| BS-q4:(14.9-27.2] | 160 / 1581 (10.1%) | 1.62 (1.22 to 2.14) | 1.32 (0.98 to 1.79) |
| **SO_2_ + NO_2_** |  |  |  |
| **Continuous** |  |  |  |
| NO_2_ | 610 / 7529 (8.1%) | 1.03 (0.89 to 1.2) | 0.97 (0.81 to 1.16) |
| SO_2_ | 610 / 7529 (8.1%) | 1.27 (1.13 to 1.42) | 1.05 (0.91 to 1.22) |
|  |  |  |  |
| **Quartiles** |  |  |  |
| NO_2_-q1:[18.2-31.4] | 62 / 1974 (3.1%) | 1 | 1 |
| NO_2_-q2:(31.4-44.4] | 142 / 1909 (7.4%) | 0.89 (0.62 to 1.28) | 0.77 (0.52 to 1.14) |
| NO_2_-q3:(44.4-46.6] | 224 / 1868 (12.0%) | 0.88 (0.59 to 1.33) | 0.72 (0.46 to 1.12) |
| NO_2_-q4:(46.6-67.9) | 182 / 1778 (10.2%) | 1.01 (0.68 to 1.50) | 0.87 (0.56 to 1.34) |
| SO_2_-q1:[6.5-29.2] | 60 / 1974 (3.0%)) | 1 | 1 |
| SO_2_-q2:(29.2-33.0] | 113 / 1820 (6.2%) | 1.07 (0.74 to 1.53) | 0.93 (0.63 to 1.37) |
| SO_2_-q3:(33.0-41.2] | 213 / 2069 (10.3%) | 1.67 (1.17 to 2.39) | 1.30 (0.89 to 1.92) |
| SO_2_-q4:(41.2-57.3) | 224 / 1666 (13.4%) | 1.81 (1.27 to 2.58) | 1.17 (0.78 to 1.76) |

Supplemental Table 2: Single-pollutant hazard ratios (HR) and 95% confidence intervals (CI), per increase of 10 µg/m^3^ for the continuous variables, between air pollution exposure to BS, SO_2_ and NO_2_ and CVD mortality and morbidity (1989-2011) in SABRE. Fully-adjusted model: model adjusted for cohort, age, gender diabetes, smoking status, ethnicity, type of employment, 1991 Carstairs index and baseline CVD.

|  | **# events / n (%)** | **Fully-adjusted model** |
| --- | --- | --- |
| **Continuous** |  |  |
| NO_2_ | 1412 / 4050 (34.9%) | 0.97 (0.81 to 1.16) |
| SO_2_ | 1412 / 4050 (34.9%) | 1.05 (0.91 to 1.22) |
| BS | 1412 / 4050 (34.9%) | 1.11 (0.76 to 1.61) |
|  |  |  |
| **Quartiles** |  |  |
| NO_2_-q1:[22.1-44.6] | 381 / 1015 (37.5%) | 1 |
| NO_2_-q2:(44.6-45.8] | 426 / 1073 (39.7%) | 1.15 (0.99 to 1.32) |
| NO_2_-q3:(45.8-48.8] | 345 / 952 (36.2%) | 1.09 (0.94 to 1.27) |
| NO_2_-q4:(48.8-63.0) | 260 / 1010 (25.7%) | 0.91 (0.76 to 1.10) |
| SO_2_-q1:[16.4-32.9] | 276 / 1117 (24.7%)) | 1 |
| SO_2_-q2:(32.9-38.5] | 371 / 1051 (35.3%) | 1.19 (1.00 to 1.42) |
| SO_2_-q3:(38.5-44.2] | 398 / 978 (40.7%) | 1.14 (0.94 to 1.38) |
| SO_2_-q4:(44.2-52.5) | 367 / 904 (40.6%) | 1.14 (0.94 to 1.37) |
| BS-q1:[1.31-11.4] | 254 / 1015 (25.0%) | 1 |
| BS-q2:(11.4-12.9] | 394 / 1138 (34.6%) | 1.16 (0.97 to 1.38) |
| BS-q3:(12.9-14.9] | 440 / 1111 (39.6%) | 1.08 (0.89 to 1.30) |
| BS-q4:(14.9-19.4] | 324 / 786 (41.2%) | 1.12 (0.92 to 1.36) |

Supplemental Table 3: Single-pollutant hazard ratios (HR) and 95% confidence intervals (CI), per increase of 10 µg/m^3^ for the continuous variables, between between ESCAPE air pollution estimates of NO_2_, NO_x_, PM_10_, PM_coarse_, PM_2.5_ and CVD mortality and morbidity (1989-2011) in SABRE. Fully-adjusted model: model adjusted for cohort, age, gender diabetes, smoking status, ethnicity, type of employment, 1991 Carstairs index and baseline CVD.

|  | **# events / n (%)** | **Fully-adjusted model** |
| --- | --- | --- |
| **Continuous** |  |  |
| NO_2_ | 1233 / 3682 (33.5%) | 0.96(0.89 to 1.05) |
| NO_x_ | 1233 / 3682 (33.5%) | 0.98(0.94 to 1.01) |
| PM_10_ | 1233 / 3682 (33.5%) | 0.97(0.72 to 1.31) |
| PM_coarse_ | 1233 / 3682 (33.5%) | 1.01(0.56 to 1.82) |
| PM_2.5_ | 1233 / 3682 (33.5%) | 0.54(0.26 to 1.13) |
|  |  |  |
| **Quartiles** |  |  |
| NO_2_-q1:[12.9-28.4] | 297 / 933 (31.8%) | 1 |
| NO_2_-q2:(28.4-30.7] | 332 / 915 (36.3%) | 1(0.84 to 1.18) |
| NO_2_-q3:(30.7-32.6] | 345 / 933 (37.0%) | 1.05(0.89 to 1.24) |
| NO_2_-q4:(32.6-91.8) | 259 / 901 (28.7%) | 0.95(0.8 to 1.14) |
| NO_x_-q1:[19.7-40.9] | 303 / 922 (32.9%)) | 1 |
| NO_x_-q2:(40.9-46.8] | 304 / 920 (33.0%) | 0.88(0.75 to 1.03) |
| NO_x_-q3:(46.8-51.6] | 336 / 921 (36.5%) | 0.95(0.81 to 1.12) |
| NO_x_-q4:(51.6-242) | 290 / 919 (31.6%) | 0.94(0.79 to 1.11) |
| PM_10_-q1:[11.8-16.5] | 298 / 969 (30.8%) | 1 |
| PM_10_-q2:(16.5-17.3] | 296 / 893 (33.1%) | 1.03(0.87 to 1.21) |
| PM_10_-q3:(17.3-18.5] | 346 / 930 (37.2%) | 1.05(0.89 to 1.25) |
| PM_10_-q4:(18.5-25.8] | 293 / 890 (32.9%) | 1(0.85 to 1.18) |
| PM_coarse_-q1:[5.6-6.3] | 335 /1094 (30.6%) | 1 |
| PM_coarse_-q2:(6.3-6.8] | 229 / 747 (30.6%) | 1.05(0.88 to 1.24) |
| PM_coarse_-q3:(6.8-7.3] | 374 / 938 (39.9%) | 1.24(1.06 to 1.46) |
| PM_coarse_-q4:(7.3-10.8] | 295 / 903 (32.7%) | 1.03(0.88 to 1.21) |
| PM_2.5_-q1:[8.2-9.6] | 331 / 960 (34.5%) | 1 |
| PM_2.5_-q2:(9.6-10.0] | 315 / 911 (34.6%) | 0.99(0.84 to 1.16) |
| PM_2.5_-q3:(10.0-10.5] | 338 / 1009 (33.5%) | 0.92(0.78 to 1.07) |
| PM_2.5_-q4:(10.5-16.5] | 249 / 802 (31.0%) | 0.92(0.77 to 1.09) |

Supplemental Table 4: Single-pollutant hazard ratios (HR) and 95% confidence intervals (CI), per increase of 10 µg/m^3^ for the continuous variables, between air pollution exposure to BS, SO_2_, NO_2_ (contemporaneous 1991 estimates) and CVD mortality (1989-2015). Comparison of results between competing risk regression modelling and Cox modelling. M1: model adjusted only for cohort to which the participant belongs. M2: M1 + age, gender. M3: M2 + type of employment, 1991 Carstairs index. M4 (fully-adjusted model): M3 + diabetes, smoking status, ethnicity, baseline CVD.

|  | **M1** | **M2** | **M3** | **M4** |
| --- | --- | --- | --- | --- |
| **NO_2_** |  |  |  |  |
| Competing risk | 1.03 (0.90 to 1.18) | 1.06 (0.91 to 1.23) | 0.94 (0.79 to 1.12) | 0.97 (0.81 to 1.16) |
| Cox | 1.04 (0.89 to 1.21) | 1.06 (0.90 to 1.25) | 0.94 (0.78 to 1.12) | 0.96 (0.80 to 1.15) |
| **SO_2_** |  |  |  |  |
| Competing risk | 1.27 (1.13 to 1.42) | 1.19 (1.05 to 1.34) | 1.07 (0.94 to 1.23) | 1.05 (0.91 to 1.22) |
| Cox | 1.27 (1.13 to 1.43) | 1.18 (1.04 to 1.33) | 1.06 (0.93 to 1.21) | 1.05 (0.91 to 1.21) |
| **BS** |  |  |  |  |
| Competing risk | 1.66 (1.19 to 2.32) | 1.44 (1.02 to 2.04) | 1.16 (0.81 to 1.65) | 1.11 (0.76 to 1.61) |
| Cox | 1.66 (1.21 to 2.27) | 1.42 (1.03 to 1.95) | 1.13 (0.81 to 1.57) | 1.09 (0.77 to 1.55) |

Supplemental Table 5: Single-pollutant hazard ratios (HR) and 95% confidence intervals (CI), per increase of 10 µg/m^3^ for the continuous variables, between ESCAPE 2010-11 air pollution estimates of NO_2_, NO_x_, PM_10_, PM_coarse_, PM_2.5_ and CVD mortality (1989-2015). Comparison of results between competing risk regression modelling and Cox modelling. M1: model adjusted only for cohort to which the participant belongs. M2: M1 + age, gender. M3: M2 + type of employment, 1991 Carstairs index. M4 (fully-adjusted model): M3 + diabetes, smoking status, ethnicity, baseline CVD.

|  | **M1** | **M2** | **M3** | **M4** |
| --- | --- | --- | --- | --- |
| **NO_2_** |  |  |  |  |
| Competing risk | 1.06 (0.93 to 1.20) | 1.10 (0.96 to 1.26) | 1.00 (0.86 to 1.17) | 1.03 (0.89 to 1.20) |
| Cox | 1.06 (0.93 to 1.22) | 1.12 (0.97 to 1.29) | 1.02 (0.87 to 1.18) | 1.04 (0.90 to 1.21) |
| **NO_x_** |  |  |  |  |
| Competing risk | 1.01 (0.96 to 1.07) | 1.03 (0.97 to 1.09) | 1.00 (0.93 to 1.07) | 1.01 (0.94 to 1.07) |
| Cox | 1.01 (0.95 to 1.07) | 1.04 (0.98 to 1.10) | 1.00 (0.94 to 1.07) | 1.01 (0.95 to 1.08) |
| **PM_10_** |  |  |  |  |
| Competing risk | 1.28 (0.82 to 2.01) | 1.36 (0.86 to 2.15) | 1.12 (0.68 to 1.85) | 1.16 (0.70 to 1.92) |
| Cox | 1.29 (0.79 to 2.09) | 1.40 (0.86 to 2.30) | 1.14 (0.68 to 1.91) | 1.13 (0.68 to 1.90) |
| **PM_coarse_** |  |  |  |  |
| Competing risk | 1.02 (0.40 to 2.60) | 1.12 (0.43 to 2.91) | 0.82 (0.29 to 2.33) | 0.89 (0.31 to 2.55) |
| Cox | 1.03 (0.38 to 2.76) | 1.19 (0.44 to 3.20) | 0.85 (0.30 to 2.42) | 0.90 (0.32 to 2.54) |
| **PM_2.5_** |  |  |  |  |
| Competing risk | 1.52 (0.52 to 4.46) | 2.10 (0.69 to 6.44) | 1.15 (0.35 to 3.81) | 1.30 (0.39 to 4.34) |
| Cox | 1.52 (0.50 to 4.69) | 2.28 (0.72 to 7.22) | 1.20 (0.36 to 4.02) | 1.17 (0.35 to 3.88) |

Supplemental Table 6: Single-pollutant hazard ratios (HR) and 95% confidence intervals (CI), per increase of 10 µg/m^3^ for the continuous variables, between air pollution exposure to BS, SO_2_, NO_2_ (contemporaneous 1991 estimates), between ESCAPE 2010-11 air pollution estimates of NO_2_, NO_x_, PM_10_, PM_coarse_, PM_2.5_, and CVD mortality (1989-2015). M4 (fully-adjusted model): model adjusted for cohort, age, gender, type of employment, 1991 Carstairs index, diabetes, smoking status, ethnicity, baseline CVD.

|  | **M4** | **M4 minus adjustment for diabetes and baseline CVD** |
| --- | --- | --- |
| **Contemporaneous 1991 estimates** |  |  |
| NO_2_ | 0.97 (0.81 to 1.16) | 0.99 (0.83 to 1.17) |
| SO_2_ | 1.05 (0.91 to 1.22) | 1.03 (0.89 to 1.19) |
| BS | 1.11 (0.76 to 1.61) | 1.11 (0.76 to 1.61) |
|  |  |  |
| **ESCAPE 2010-11 estimates** |  |  |
| NO_2_ | 1.03 (0.89 to 1.20) | 1.03 (0.89 to 1.21) |
| NO_x_ | 1.01 (0.94 to 1.07) | 1.01 (0.94 to 1.08) |
| PM_10_ | 1.16 (0.70 to 1.92) | 1.16 (0.70 to 1.91) |
| PM_coarse_ | 0.89 (0.31 to 2.55) | 0.88 (0.31 to 2.50) |
| PM_2.5_ | 1.30 (0.39 to 4.34) | 1.27 (0.38 to 4.28) |
